# The Field of Science

**Published:** 1893-12-09

**Authors:** 


### The Field of Science.

The army medical staff are to be afforded opportunities next summer of a more definitely practical knowledge of their field duties during war. We had Mr. Campbell Bannerman's assurances not long since in the House of Commons, that steps will be taken for a fortnight's practical instruction in what he rightly observes is the most essential and necessary duty that the medical services can be called upon to perform. Up to now officers and men of the English Army Medical Corps have not been on a sufficiently intimate footing with the machinery, so to speak, of field hospitals and their equipment, and we are glad to learn that a practical initiation into such duties will supplement the former more theoretical one.

We are reminded in an interesting article which appeared recently in the pages of a weekly contemporary of the debatable ground which yet exists regarding the physiological effect produced by inhaling pure oxygen gas into the lungs. The Committee of the British Association reported recently on the results accruing from the use of this gas in cases of suffocation, regarded with special reference to its application in asphyxia of coal miners, and the conclusion arrived at was that the gas was practically of no more effect in experiments which had been tried on asphyxiated rabbits than ordinary air. And this was stated to be the case, whether such rabbits had been artificially assisted to recovery or not, and whether the process of restoration took place in an atmosphere free from carbonic acid gas, or in one contaminated by it.

It was also found, on the authority of the same report, that inhaled by a healthy man for five minutes, oxygen produced no appreciable effect on pulse or breathing, and whether pure or diluted, the gas had similarly no effect on a patient suffering from difficulty of breathing connected with heart affection. Our correspondent goes on to point out that the evidence quoted above is not exactly in correspondence with much that has previously existed. The time has, therefore, come when a more definite and exhaustive research should be undertaken regarding the exact place oxygen may be expected to play in the hands of physicians, seeing that up to now its value has been vaunted and chronicled as the result of experience.

There has been of late years so much discussion and disagreement concerning the fees of medical men in Russia that at last the Government has taken the matter in hand, and has settled a certain specialised scale of charges which doctors will in future be entitled to make in that country. Medical fees will now be chargeable in proportion to the income of the patients, and districts and classes have been duly classified in view of this new regulation.

We gather from *L'Elettricisita* that the great difficulty which seismologists have hitherto felt in ascertaining the exact time at which an earthquake shock strikes a given locality has been at length obviated. An ingenious electrical instrument has been devised consisting of a chronometer, which is photographed by "the light of an incandescent lamp, lighted for about a quarter of a second by a current established automatically through the effect of the shock." So far the new seismograph has worked well in practice, and inasmuch as it is the outcome of a definite scientific principle, it is well calculated to be a sure time-reckoner.

The habit of opium smoking has attained considerable dimensions in Australia; and Victoria, following the tradition of paternal government, has been the first of the colonies to take the matter into consideration. A Bill is at present before the Legislature with the following regulations as to the importation and use of opium. Licences must be obtained for the importation of the drug, and it may only in future be used for medicinal purposes. No ship is to be allowed to enter any Victorian port with more than 50lb. weight of opium on board, under penalties ranging from £25 to £500. Importers will only be permitted to sell this to medical practitioners, chemists, members of the College of Veterinary Surgeons, or licensed persons, and records are ordered to be kept of the disposal of all importations. The use of opium, except for medicinal purposes, is made henceforth a penal offence, and the growing or manufacturing of the same is likewise interdicted in Victoria. It will further be held in the power of the Governor in Council to prohibit the importation or sale of any medicine which contains an undue proportion of the drug, and full power is granted for making all necessary regulations to annihilate the opium-smoking evil now existent in the colony.

